# TIPSS as a bridge to extrahepatic abdominal surgery: a case report

**DOI:** 10.1093/omcr/omac029

**Published:** 2022-04-19

**Authors:** Audrey Pamela Kapeleris, Suresh Venkatachalapathy

**Affiliations:** Queen’s Medical Centre, Nottingham University Hospitals NHS Trust, Nottingham, UK; Nottingham Digestive Diseases Centre (NDDC) and NIHR Nottingham Biomedical Research Centre (BRC), Nottingham University and Nottingham University Hospitals NHS Trust, Queen’s Medical Centre, Nottingham, UK

## Abstract

Severe portal hypertension in cirrhosis is a relative contraindication to major surgical intervention. Pre-surgical placement of a transjugular intrahepatic portosystemic shunt (TIPSS) can potentially reduce portal hypertension and the risk of intraoperative bleeding. Two patients in our service, with cirrhosis and portal hypertension, required abdominal surgery and underwent TIPSS placement as a potential bridging therapy. Patient 1, a 56-year-old female, successfully underwent surgery with no intraoperative complications. Patient 2, a 36-year-old male, experienced liver decompensation post-TIPSS and is currently awaiting a liver and bowel transplant. Prophylactic TIPSS placement may allow some patients with decompensated cirrhosis to successfully undergo major extrahepatic abdominal surgery. However, careful patient selection and preoperative counselling for decompensation is necessary.

## INTRODUCTION

More than 10% of patients with cirrhosis will require surgery in the last 2 years of life [1]. Despite progress in perioperative management and surgical techniques, extrahepatic surgery morbidity and mortality can be as high as 50% in these patients compared to ~1% in patients without cirrhosis [2].

A means of improving surgical mortality in patients with cirrhosis is via preoperative placement of a transjugular intrahepatic portosystemic shunts (TIPSS). TIPSS have been used to treat complications of portal hypertension, including bleeding varices, refractory ascites and hepatic hydrothorax. TIPSS placement is less invasive than surgical shunts and can be performed in patients with a higher degree of liver insufficiency [3].

Evidence for preoperative TIPSS placement is still limited and with often contradictory findings. Several large retrospective studies found that certain patients with cirrhosis can safely undergo major surgery after successful portal decompression via TIPSS, while the only comparative study did not observe any improved survival between control and treatment groups [4]. We present two patients who underwent TIPSS as a bridge procedure and their outcomes.

## CASE REPORT

Clinical and serological data for all patients pre- and post-TIPSS are summarized in Table 1.

**Table 1:** Summary of patient demographics, Child-Pugh scores, MELD scores, pre- and post-TIPSS HVPG values and outcomes for all four patients involved in this study; Patient 1 experienced a larger drop in HVPG post-TIPSS than Patient 2

Patient	Age	Sex	Child-Pugh score	MELD score	Intended surgery	Pre-TIPSS HVPG (mmHg)	Post-TIPSS HVPG (mmHG)	Outcome
P1	56	F	CP A (6)	9	LAR	33	7	LAR successful
P2	36	M	CP B (9)	11	Total colectomy	22	19	Thrombosed TIPSS, awaiting liver and bowel transplant

### Case 1: 56-year-old female requiring lower anterior resection

Patient 1 was a 56-year-old female with sigmoid adenocarcinoma who required a lower anterior resection (LAR). Co-morbidities included diabetes, a body mass index of 43 and non-alcoholic steatohepatitis with cirrhosis and oesophageal varices. The best treatment option for Patient 1 was surgical resection following neoadjuvant chemotherapy. But her cirrhosis presented a significant mortality risk. Her Child-Pugh score was 6 and her MELD score was 9.

Preoperative TIPSS placement was suggested as a means of reducing portal hypertension and mitigating bleeding risk. As the potential benefit outweighed the risks, the patient underwent the prophylactic TIPSS treatment. Her pre-TIPSS HVPG was 33 mmHg, post-TIPSS pressure measured 7 mmHg. She successfully underwent her LAR and her recovery was uneventful.

### Case 2: 36-year-old male requiring colectomy

Patient 2 was a 36-year-old male with ulcerative colitis with dysplasia requiring a colectomy. Medical history included autoimmune hepatitis and primary sclerosing cholangitis with secondary cirrhosis and ascites. His suffered from significant portal hypertension with a Class B(9) Child-Pugh score. His MELD score was 11. He underwent a TIPSS procedure with the goal of decompression via TIPSS placement and potentially improving the outcome of colonic surgery. His pre-TIPSS HVPG was 22 mmHg and post-TIPSS pressure measured 19 mmHg (inadequate pressure reduction). Unfortunately, he received infliximab treatment around the same time the TIPSS was placed, resulting in acute decompensation with ascites and oedema. There was stent thrombosis soon after the infliximab and TIPSS therapies, so it is likely that the treatments initiated decompensation rather than simple disease progression. Ultimately, Patient 2’s TIPSS thrombosed and he is currently on the wait list for a liver and bowel transplant.

## DISCUSSION

Cirrhotic patients with portal hypertension present a unique challenge, often having co-morbidities that complicate management [5]. High rates of surgical morbidity and mortality have been reported in patients with cirrhosis undergoing various surgical procedures [6]. Patients with cirrhosis are more susceptible to the adverse hepatic effects of general anaesthesia resulting from reduced hepatic blood flow [7]. Cirrhotic patients also have an increased risk of perioperative bleeding, sepsis, wound dehiscence and renal failure owing to underlying portal hypertension, coagulopathy and impaired reticuloendothelial cell function [8]. Our small case series shows that the trend towards decompensation is bigger if there is inadequate pressure reduction (clinical failure of TIPSS).

The body of evidence examining the effects of preoperative TIPSS placement is still quite limited even though the procedure was described over 30 years ago [9]. Vinet *et al*. (2006) found no benefit of preoperative TIPSS placement when comparing 18 patients who underwent TIPSS compared to 17 matched controls [4]. By contrast, Schmitz *et al*. (2020) found preoperative TIPSS placement allowed abdominal surgery to be performed on 11 of 21 patients [10]. And Gil *et al*. (2004) reported that pre-surgery TIPSS placement in three patients reduced portal hypertension by an average of 18 mmHg and allowed all three patients to successfully receive abdominal surgery [11].

Our two case studies show that TIPSS placement is more likely to allow for abdominal surgery if there is significant pressure reduction post-TIPSS. This means that TIPSS may allow some patients with cirrhosis and portal hypertension to successfully undergo major and complex extrahepatic abdominal surgery with reduced intraoperative and post-operative morbidity. However, the risk of decompensation and mortality following TIPSS placement suggests that careful patient selection and preoperative counselling is an absolute necessity.
